# Using Infrared Raman Spectroscopy with Machine Learning and Deep Learning as an Automatic Textile-Sorting Technology for Waste Textiles

**DOI:** 10.3390/s25010057

**Published:** 2024-12-25

**Authors:** Pei-Fen Tsai, Shyan-Ming Yuan

**Affiliations:** Department of Computer Science, National Yang Ming Chiao Tung University, ChiaoTung Campus, Hsinchu 300093, Taiwan; alicetsai.cs10@nycu.edu.tw

**Keywords:** circular economy, textile recycling, Raman spectroscopy, artificial intelligence, machine learning, neural networks, textile sorting

## Abstract

With the fast-fashion trend, an increasing number of discarded clothing items are being eliminated at the stages of both pre-consumer and post-consumer each year. The linear economy produces large volumes of waste, which harm environmental sustainability. This study addresses the pressing need for efficient textile recycling in the circular economy (CE). We developed a highly accurate Raman-spectroscopy-based textile-sorting technology, which overcomes the challenge of diverse fiber combinations in waste textiles. By categorizing textiles into six groups based on their fiber compositions, the sorter improves the quality of recycled fibers. Our study demonstrates the potential of Raman spectroscopy in providing detailed molecular compositional information, which is crucial for effective textile sorting. Furthermore, AI technologies, including PCA, KNN, SVM, RF, ANN, and CNN, are integrated into the sorting process, further enhancing the efficiency to 1 piece per second with a precision of over 95% in grouping textiles based on the fiber compositional analysis. This interdisciplinary approach offers a promising solution for sustainable textile recycling, contributing to the objectives of the CE.

## 1. Introduction

**Figure 1:**
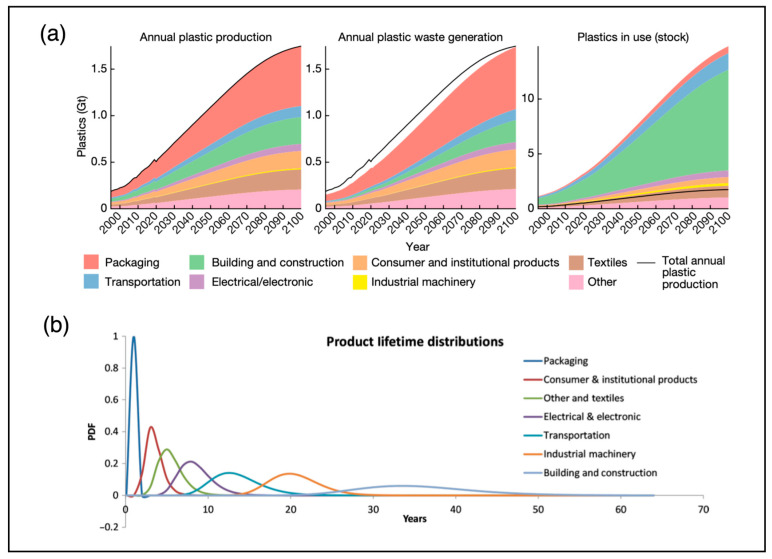
Global plastic demand and life cycle by sector. (**a**) Global plastic production, waste, and in-use volume projections from 2000 to 2100 (Stegmann et al., 2022) [1]. (**b**) Product life cycle by sector (Geyer, R. et al., 2017) [2].

The projection of global plastic production from 2000 to 2100 shows that plastic waste generation will increase drastically, reaching over 15 billion metric tons by 2100, as shown in Figure 1a [1]. The textile sector is the second-largest contributor to this increase. With the high demand for fast fashion and the lack of an effective solution to reduce, reuse, and recycle [2], textiles tend to have a short product life. As shown in Figure 1b, it is difficult to reduce product manufacturing effectively to meet consumer demand. Consequently, there has been an increasing number of studies on developing waste-textile-recycling technology to extend the textile life cycle, in recent years.

**Figure 2:**
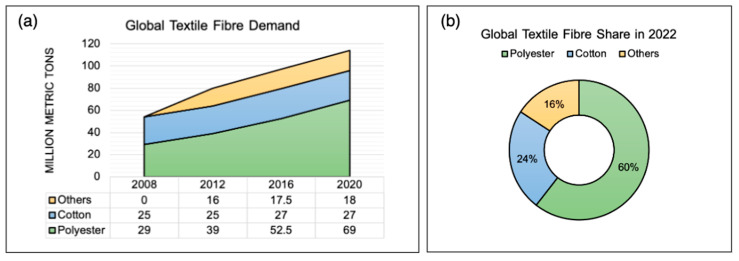
The trends and shares of the global textile demands. (**a**) Global textile demands for polyester, cotton, and others. (**b**) Global textile demand share percentages in 2020 (redrawn based on [3]).

**Figure 3:**
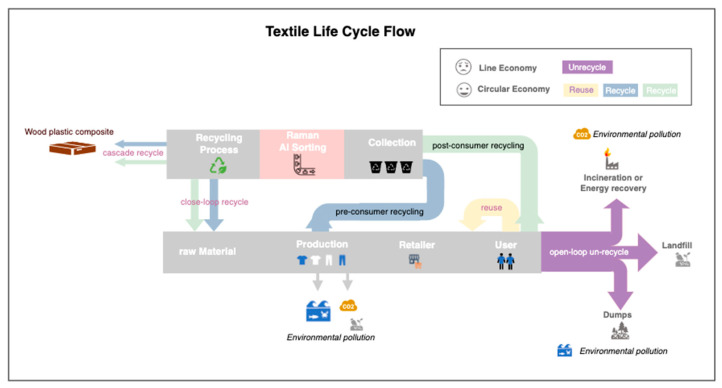
The textile life-cycle flow includes un-recycling, reuse, and closed-loop and open-loop recycling. The un-recycling of waste textiles is in the linear economy, while the 2Rs of reuse and recycling are in the circular economy (redrawn from (MDBC, 2021) [3]).

In 2020, in the global textile industry report [3], there was a polyester (PES) fiber demand of 69 million metric tons, representing 60% of the market share, which highlights the highest textile demand and an increasing trend, as seen in Figure 2a,b. Consumers frequently dispose of old clothing, leading to textile waste that often ends up in landfills or incinerators, causing land pollution and contributing to greenhouse gas emissions, as shown in Figure 3 (purple flow). To achieve environmentally sustainable governance (ESG), implementing the 3Rs—reuse, reduction, and recycling—is critical for extending the product life cycle. To shift the flow from purple (unrecycled) to blue and green (recycled) materials (Figure 3), an efficient and accurate AI-based sorting system is essential for both open-loop and closed-loop recycling.

### 1.1. Textile Recycling and the Circular Economy

**Figure 4:**
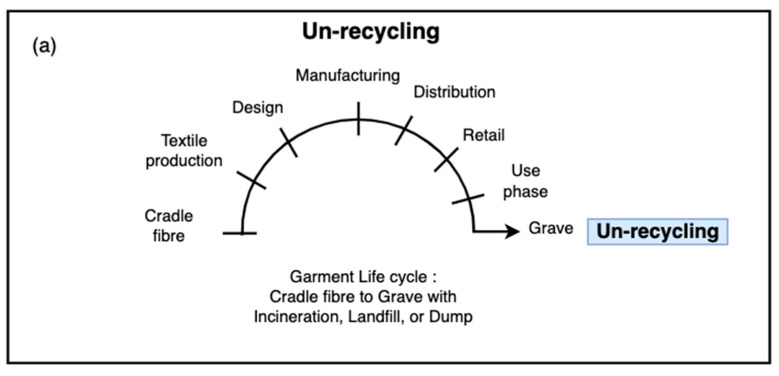

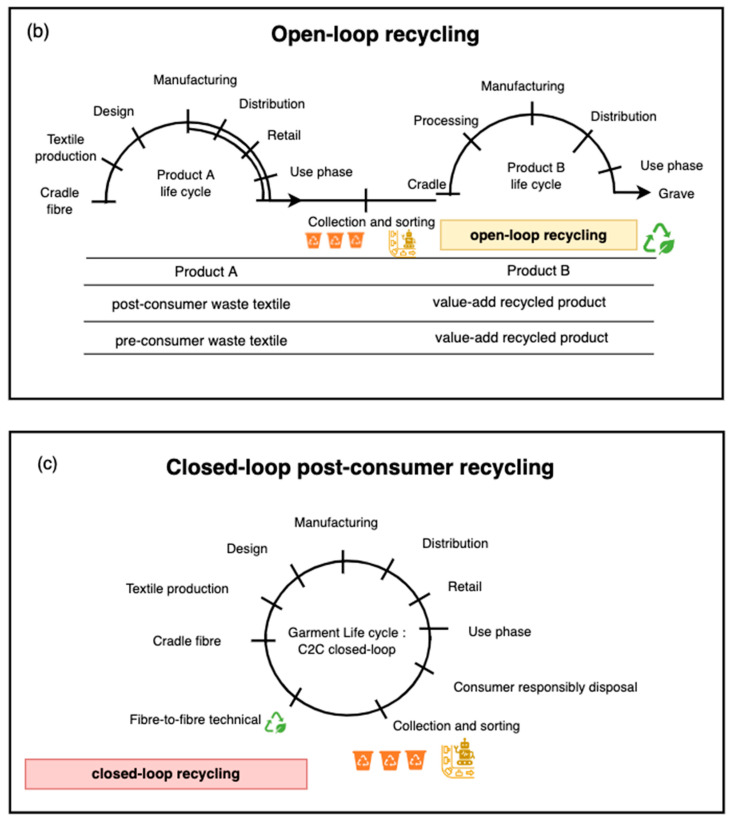
A PET textile’s life cycle (Redrawn from (A. Payne et al., 2015)) [4]. (**a**) Un-recycling a PET garment from cradle to grave. (**b**) Open-loop recycling in a PET garment’s life cycle. (**c**) Closed-loop recycling in a PET garment’s life cycle.

The textile life cycle can be classified into four modes, as defined by A. Payne [4] and illustrated in Figure 4, as: un-recycling (Figure 4a), open-loop recycling (Figure 4b), and closed-loop recycling (Figure 4c).

Open-loop recycling (OLP), also called “downcycling”, occurs at both the pre-consumer and post-consumer stages and is relatively easier to achieve compared to closed-loop recycling. In contrast, closed-loop recycling involves reprocessing polyester textiles, enabling them to reenter the same production cycle through chemical recycling. This process maintains high product value and aligns with circular economy (CE) principles, which emphasize the elimination of waste and pollution, the circulation of products and materials, and the regeneration of nature.

The transition toward a circular economy directly aligns with Sustainable Development Goal (SDG) 12: Responsible Consumption and Production. By implementing closed-loop recycling and fostering innovative textile-sorting technologies, the environmental impact of PET textiles can be minimized.

### 1.2. Challenges in Achieving a Circular Economy in the Textile Industry

**Figure 5:**
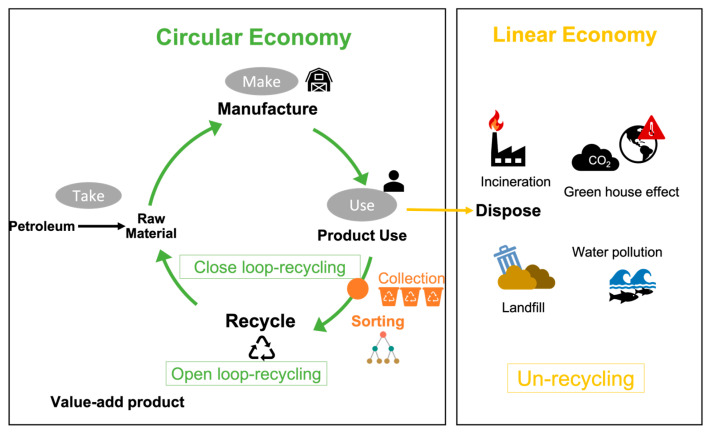
Adding waste textile collection and sorting to the production is critical for moving from a linear economy to a circular economy.

One of the primary challenges for achieving a circular economy in the textile industry is the lack of efficient sorting methods because of complex fiber combinations in waste textiles [5]. Unlike plastic packaging waste, textiles consist of varying material blends, complicating the recycling process. In addressing this issue, our study focuses on SDG 9: Industry, Innovation, and Infrastructure, by developing advanced artificial-intelligence-based sorting models. At Taiwan’s Institute of Industrial Technology Research (ITRI), we successfully created a highly accurate Raman-spectroscopy-based textile sorter. This innovation enables the precise sorting of textile materials, facilitating the transformation of PET textile waste from a linear economy to a sustainable circular economy. This approach can mitigate water pollution, minimize greenhouse gas emissions, and extend the product life cycle, as shown in Figure 5.

Achieving these goals requires collaboration across sectors, countries, and organizations, emphasizing SDG 17: Partnerships for the Goals. Partnerships among policymakers, researchers, industries, and environmental organizations are essential for driving innovation and fostering a global commitment to sustainable textile management. By integrating advanced technologies, like AI-based sorting, industries can collaboratively promote a circular economy while protecting the environment and meeting the rising demand for sustainable materials.

### 1.3. Vibrational Spectroscopy and AI for Textile Sorting

**Figure 6:**
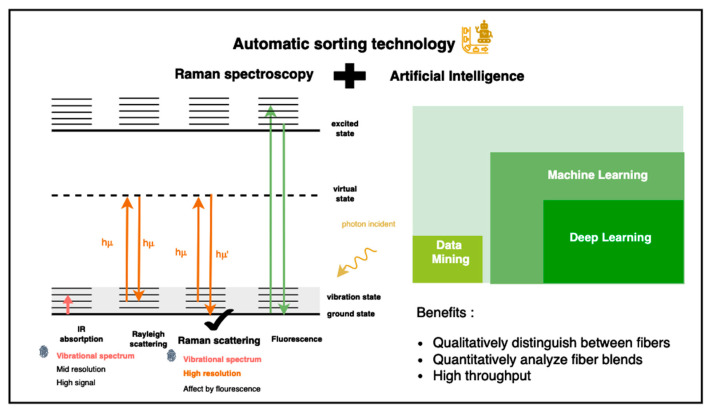
The high-efficiency automatic textile-sorting technology combines Raman spectroscopy and artificial intelligence technologies of data mining, machine learning, and deep learning.

Vibrational spectroscopy [6], including near-infrared (NIR) [7] and Raman techniques [8,9,10], is widely used to analyze molecular compositions. NIR spectroscopy relies on vibrational bond absorption (from 780 nm to 2526 nm), while Raman spectroscopy detects vibrational states through inelastic scattering. We utilize informative Raman spectra as a vibrational spectrum for analyzing textiles’ compositions, which serve as chemical fingerprints. We combine the AI model with Raman spectroscopy for qualitative and quantitative fiber analysis to classify fibers into purer categories and increase the flow of closed-loop recycling, as shown in Figure 6.

### 1.4. Importance of Sorting for rPET Recycling

Polyester, a petroleum-based synthetic fiber, remains in high demand. The recyclability potential index (RPI) [4] ranks PES first, for its environmental and economic advantages, among ten commonly used fibers, as reported by Muthu et al. [11]. This ranking highlights its recyclability potential for ecological sustainability.

Efficient textile sorting is essential for successful closed-loop recycling, particularly for polyester textiles, as it ensures higher purity in material streams. Accurate sorting enables pure polyester (PES) and cotton (CO) fibers to enter the recycling process, producing high-quality recycled polyethylene terephthalate (rPET). Improperly sorted or low-purity materials result in contamination, limiting high-value recycling and often leading to downcycling or disposal.

Sorting textiles into six distinct classes improves the efficiency of the recycling process by separating materials with different properties, enabling the use of the most suitable recycling method for each category.

### 1.5. Sorting and Recycling Processes for Different Textile Classes

**Figure 7:**
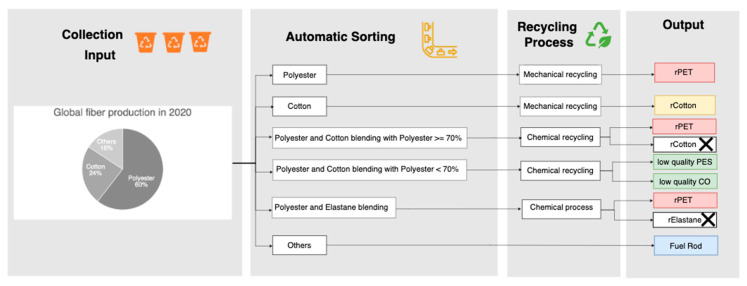
Waste textile collection, sorting, and recycling via mechanical and chemical processes for pure-composition textiles and blended textiles, as well as the outputs after recycling.

The classification of textiles into six categories is essential for optimizing the recycling process in Figure 7. By sorting textiles based on fiber composition and material content, each category can be treated using the most effective recycling method. This ensures higher purity, better material recovery, and efficient closed-loop recycling. The six categories are based on the materials’ compositions, which determine the suitable recycling technique and the quality of the recycled output [12,13,14,15,16].

#### 1.5.1. 100% Cotton (CO)

- Reason for Sorting: Cotton is a natural fiber that can be efficiently recycled when not contaminated by synthetic materials. Sorting cotton ensures higher purity during recycling, leading to better-quality recycled cotton yarns or fibers;
- Recycling Method: Mechanical Recycling;
- Process: Cotton is shredded, cleaned, carded, and spun into new yarns for reuse;
- Recycling Outcome: High-quality recycled cotton yarns for new textile production.

#### 1.5.2. 100% Polyester (PES)

- Reason for Sorting: Pure polyester can be recycled more efficiently compared to blends. The absence of cotton or other materials ensures that the recycling process can focus on re-polymerizing the polyester into rPET, maintaining its properties for reuse in high-quality textile products;
- Recycling Method: Mechanical Recycling;
- Process: Polyester textiles are shredded, cleaned, carded, and spun into yarns for reuse;
- Recycling Outcome: High-quality recycled polyester (rPET).

#### 1.5.3. Polyester–Cotton Blends (PES/CO ≥ 70%)

- Reason for Sorting: Polyester–cotton blends with a higher percentage of polyester (≥70%) are common in the market because of their cost effectiveness and durability. Sorting these textiles allows for the efficient separation of polyester and cotton for recycling, enabling a higher yield of recycled polyester for rPET production;
- Recycling Method: Chemical Recycling;
- Separation Methods:
- Polyester Dissolution: Chemicals like DMSO dissolve polyester, leaving cotton intact. The polyester is recovered, purified, and re-polymerized into rPET;
- Cellulose Degradation: Cotton is broken down using hydrolysis, leaving behind pure polyester for rPET production;
- Recycling Outcome: High-quality rPET and cellulose for reuse.

#### 1.5.4. Polyester–Cotton Blends (PES/CO < 70%)

- Reason for Sorting: Blends with less than 70% polyester are more challenging to recycle because of the increased presence of cotton. Sorting them separately ensures that a more appropriate recycling method is applied to maximize material recovery. This group often requires additional chemical or combined methods to effectively separate the fibers;
- Recycling Method: Mechanical or Combined Recycling;
- Process: Textiles are shredded and treated with chemical processes to separate polyester and cotton fibers;
- Recycling Outcome: Lower-quality recycled materials, potentially requiring virgin fibers to enhance product performance.

#### 1.5.5. Polyester with Elastane (PES/EA)

- Reason for Sorting: Elastane is typically added in small quantities (<5%) to enhance the elastic properties of textiles, improving stretchability and comfort. Sorting these textiles ensures that the elastane content is taken into account when applying recycling methods, thereby preventing the contamination of recycled polyester and maintaining the quality of the final product;
- Recycling Method: Solvent-Based Recycling or Mechanical Shredding;
- Process:
  ○ Solvent-Based Techniques: Selective solvents dissolve polyester while isolating elastane for further treatment;
  ○ Mechanical Shredding: Elastane-contaminated polyester can be shredded into fibers, but this reduces the quality of recycled materials;
- Recycling Outcome: Recycled polyester with minimal elastane contamination, ensuring better-quality rPET.

#### 1.5.6. Others: Blends with Polyamide (PA) or Other Fibers (Three or More Materials)

- Reason for Sorting: Textiles containing polyamide (PA), nylon, or three or more materials (such as polyester mixed with elastane, cotton, and polyamide) are highly complex and difficult to recycle. Sorting these textiles allows for the identification of the most appropriate recycling method for each fiber type, whether it involves downcycling or valorization to value-added products;
- Recycling Method: Downcycling or Valorization;
- Process: Textiles that cannot be efficiently separated are often downcycled to lower-quality products, like wood–plastic composites and insulation materials, or used for energy recovery;
- Recycling Outcome: Limited reuse and potential disposal if not sorted correctly.

### 1.6. Valorization and the Circular Economy

By incorporating automatic sorting technology, higher purity in recycling streams can be ensured, allowing for materials to be reused more efficiently and in higher-value applications. Higher purity leads to better-quality recycled outputs, such as recycled polyester (rPET), which can be reintroduced to the textile production cycle. Waste textile valorization to value-added products, like wood–plastic composites, insulation, or industrial materials, is also possible as open-loop recycling.

### 1.7. Enhancing the Circular Economy and Sustainability

**Figure 8:**
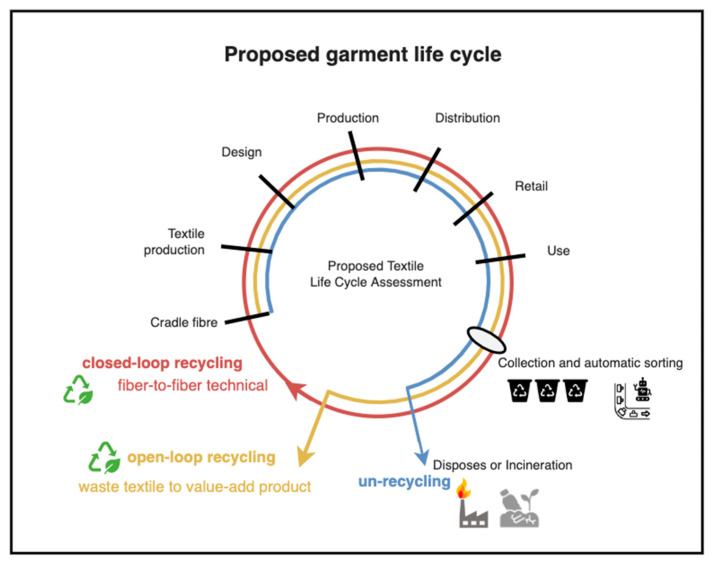
Proposed waste textile life cycle assessment with automatic sorting to achieve closed-loop recycling and open-loop recycling with extended life cycle and a comparison with un-recycling.

- Utilizing automatic sorting technology—by integrating Raman spectroscopy with AI techniques, such as data mining, machine learning, and deep learning—we can enhance the circular economy and sustainability, as shown in Figure 8, as follows:
  ○ Enhance closed-loop recycling efficiency with high-purity PET textiles;
  ○ Divert low-purity textiles to open-loop recycling, ensuring alternative uses instead of disposal;
  ○ Support waste textile valorization, turning waste into value-added products that contribute to resource sustainability;
  ○ This approach transforms the textile life cycle from an un-recycling linear economy to a circular economy, minimizing waste, reducing environmental pollution, and ensuring sustainable textile management.

## 2. Literature Review

### 2.1. Raman Spectroscopy in Fiber and Textile Analysis

Raman scattering spectroscopy was introduced by D.A. Long [8] as a robust analytical methodology for molecular vibration analysis. Symmetric molecular vibrations may be inactive in infrared spectroscopy, but they can still be observed in Raman scattering. In 1998, L.A. Lyon et al. [9] focused on Raman spectroscopy with a microscope for the biological application of surface-enhanced Raman scattering (SERS). In 2000, S.P. Mulvaney [10] reviewed Raman spectroscopy from 1997 to 1999. Since then, Raman spectroscopy has kept growing as an analytical chemical tool in various fields. In 2008, A. Kudelski [17] reviewed the applications of Raman spectroscopy from 2004 to 2008, showing that Raman measurements can create molecular vibrational spectra that act like a “fingerprint” for identifying compounds. Since then, more and more studies have used Raman spectroscopy to analyze materials like fibers, cellulose, and textiles. Notable studies include those by J.H. Wiley et al. [18] in 1987, H.G.M. Edwards et al. [19] in 1997, K. Kavkler et al. [20] in 2011, and C. Carey et al. [21] in 2015. Raman scattering provides distinct spectral patterns of textiles, helping to differentiate between pure and blended fabrics.

### 2.2. Machine-Learning and Deep-Learning Models for Raman Spectroscopy

Artificial intelligence (AI), including machine-learning (ML) and deep-learning (DL), has been used as a powerful tool in many fields, such as image processing and chemical analytical fields [22]. In Raman spectroscopy applications [23], AI models such as k-nearest neighbors (KNNs) [24], decision trees (DTs) [25], random forests (RFs) [26], support vector machines (SVMs) [27,28,29], Bayesians [30], artificial neural networks (ANNs) [31], convolutional neural networks (CNNs) [32], recurrent neural networks (RNNs) [33], and generative adversarial networks (GANs) [34] have all made significant contributions.

In the past decade, Raman spectroscopy combined with deep learning to extract spectral features for classification has proven to be effective for identifying component [35], detecting bacterial [36], and aiding medical diagnosis [37]. For environmental concerns, in 2022, E.R.K. Neo Neo [38] used Raman spectroscopy with KNNs and SVMs to sort plastic wastes. In 2020, M.K. Maruthamuthu [39] applied CNN to detect microbial contamination in water. In 2022, P.Y. Kow [40] proposed using CNN for real-time air-quality estimation based on images.

Today, many studies on using Raman spectroscopy with AI models are been published in journals focused on sustainability.

## 3. Methodology

**Figure 9:**
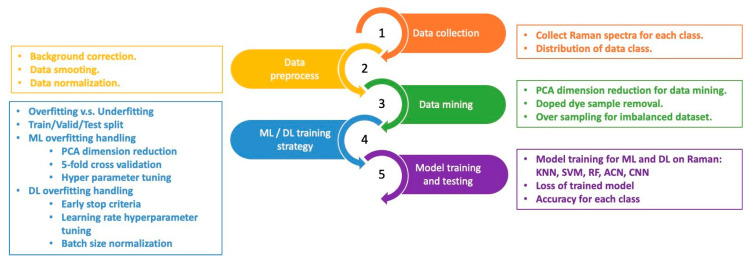
The experimental flow includes data collection, preprocessing, mining, modeling, and testing.

Due to the limited availability of waste-textile samples and dye-related fluorescence in the Raman data, we carefully manage Raman data through a series of steps. These steps include data collection, data preprocessing, data mining, machine learning (ML) or deep learning (DL) training, and model training and testing,, as shown in Figure 9.

### 3.1. Textile Labeling with FTIR Spectroscopy

The textile samples are collected from three textile factories in Taiwan. In addition to using the composition labels on the washing tags for the ground-truth labels of the samples, we use FTIR (Thermo brand) spectroscopy for compositional verification to ensure the textile compositions’ correctness. This FTIR instrument is located in the laboratory of the Material and Chemical Research Laboratories (MCLs) at the Industrial Technology Research Institute (ITRI) [41] and is calibrated annually.

### 3.2. Raman Online Hardware

**Figure 10:**
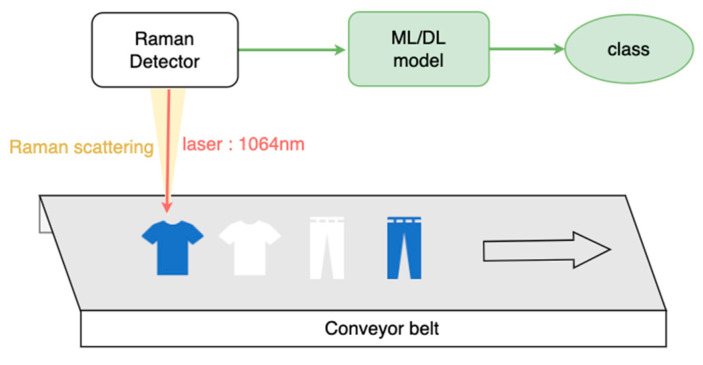
The hardware of the Raman detector and software of the automatic sorting ML/DL model (redrawn with permission from [42]).

We collect online Raman textile data from hardware at the Industrial Technology Research Institute (ITRI) (US patent: US202/013730A1) in the actual fieldwork scenario, as shown in Figure 10 [42].

- Conveyer speed: 40 cm/s;
- Camera integration time: 1 s;
- Excitation laser wavelength: 1064 nm;
- Raman spectral range: −1775~3597 cm^−1^;
- Sampling Z-height scan for signal optimization;
- Detection speed: 1 s per piece.

### 3.3. Data Collection and Label Distribution

**Figure 11:**
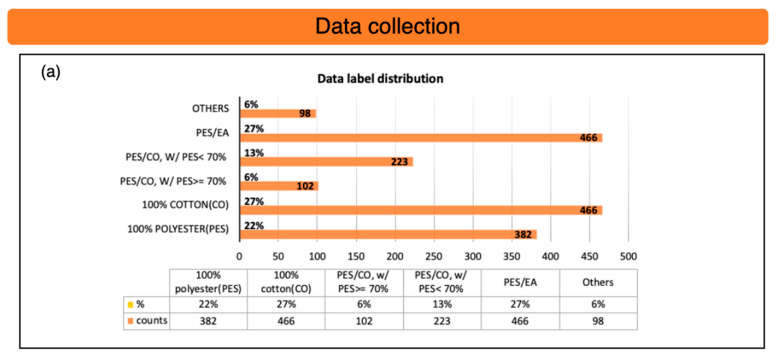

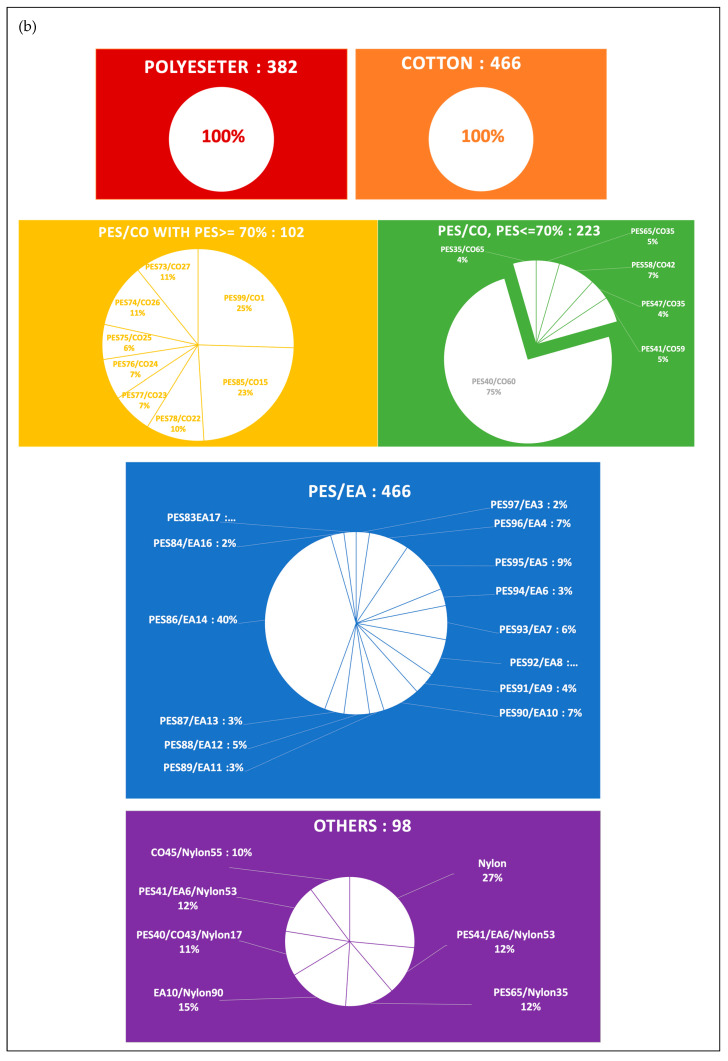
These are the Raman data for six classes. (**a**) The six classes are the distribution of the collected data. (**b**) The distribution in each class of blend combination.

A total of 225 textiles are scanned using an online Raman textile sorter, with scan taken at around ten different positions on each sample to collect Raman spectra. These samples include six types textiles: polyester (PES), cotton (CO), a polyester-cotton blend with over 70% polyester (PES/CO with PES ≥ 70%), a polyester-cotton blend with less than 70% polyester (PES/CO with PES < 70%), a polyester-elastane blend (PES/EL), and nylon or other mixed blends (Others). The overall distribution of these samples is shown, along with individual class distribution chart is shown in Figure 11a,b. The data for the classes “PES/CO with PES < 70%” and “Others” are imbalanced and need to be addressed before training the model.

### 3.4. Data Preprocessing for Fluorescence Background Reduction and Outlier Removal

**Figure 12:**
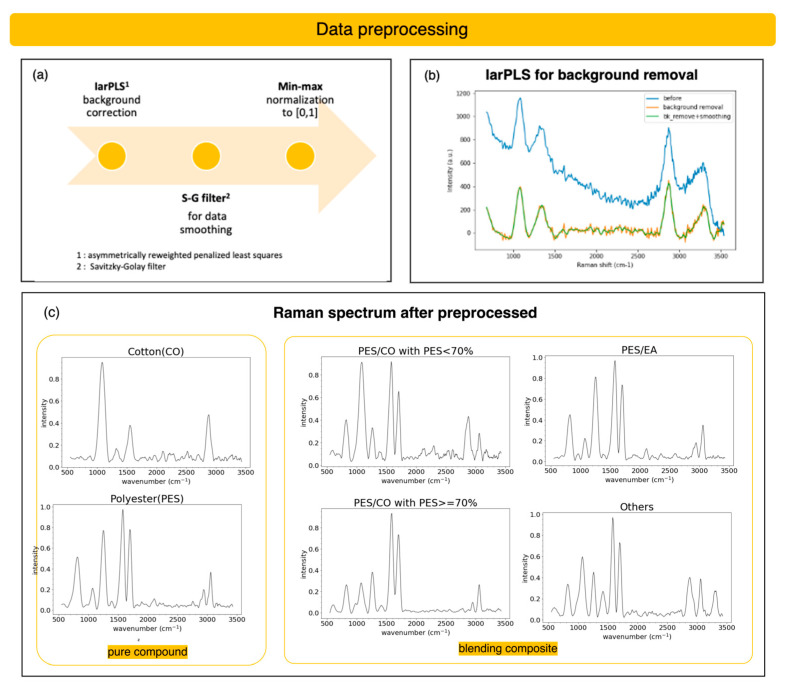
Data preprocessing of Raman spectra with background correction, S-G filtering, and intensity normalization. (**a**) The preprocess flow. (**b**) IaLS background correction before and after. (**c**) The preprocessed Raman spectra of six classes.

The dyeing of textiles is a process to transfer dyes from a solution to fiber surfaces. In that case, the laser will interact not only with textiles showing non-elastic Raman scattering but also with dyes showing a solid fluorescence background in spectra [43]. A Raman spectrum preprocessing flow is required to remove fluorescence normalization and filter out noise in spectra, as shown in Figure 12a.

To separate out the Raman signal, we perform a baseline correction with the improved asymmetrically reweighted penalized-least-squares (IarPLS) method, proposed by J. Ye et al. [44] in 2020, for Raman spectra, as shown in Figure 12b. After that, we performed the normalization after the baseline removal. In addition, to smooth the data while keeping the signal’s shape and width, we applied the Savitzky–Golay filter [45], proposed in 1964 by Savitzky and Golay in *Analytical Chemistry*, using a selected sliding window with linear least squares [46]. The filtered Raman spectrum is the final data preprocessing result. The preprocessed Raman spectra for a total of six classes are shown in Figure 12c.

### 3.5. Data Mining with PCA and Outlier Removal

**Figure 13:**
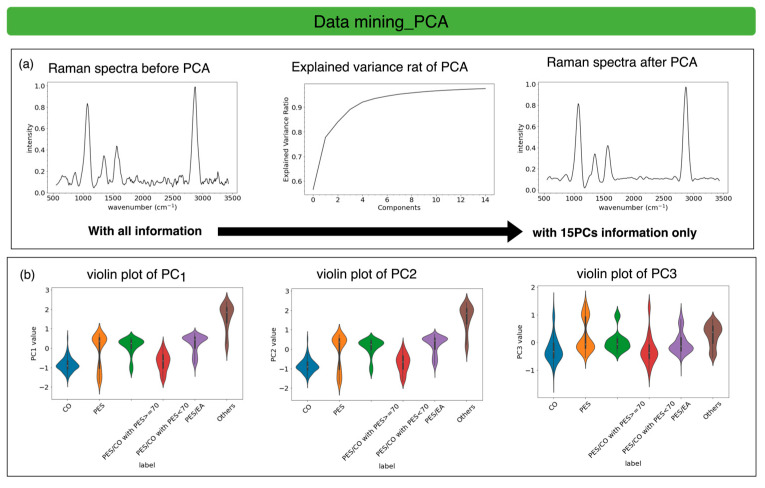
Data mining with PCA and violin plots. (**a**) Raman spectra before PCA and after PCA with PC = 15. (**b**) The violin plots for PC1, PC2, and PC3 for six classes.

The processed Raman spectra span from 560 cm^−1^ to 3430 cm^−1^ and have 342 dimensions. To visualize the data, we applied principal component analysis (PCA) to reduce the dimensionality from 342 to 15, while retaining over 95% of the original information. We, then used the 15-dimensional data to reconstruct the Raman spectrum, and shown in Figure 13a, the critical peak information was effectively preserved.

With the reduced dimensionality of 15-D, the violin plots for the top three PCs in the six classes show that between-class is separable after PCA, as shown in Figure 13b. Therefore, to achieve a higher efficiency of the online system, we introduce AI to ML and DL for feature learning. We combine PCA with ML models, including KNN, SVM, and RF, while using deep-learning models of ANN and CNN with no reduction in the dimensionality of the 342-D Raman spectrum. AI technology is introduced to learn the features of textile combinations with high-efficiency and high-accuracy classification tasks.

**Figure 14:**
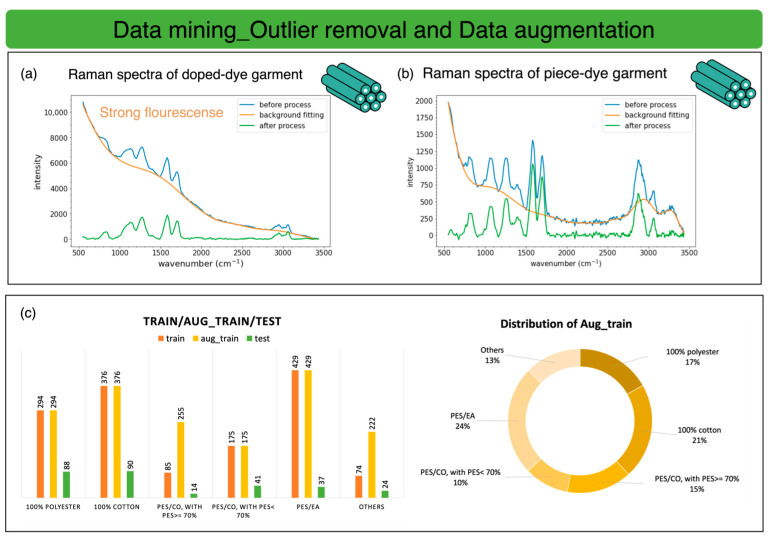
Data mining for outlier removal and data augmentation. (**a**) Raman spectra of the doped-dyed garment. (**b**) Raman spectra of the piece-dyed garment. (**c**) Training/testing split and up-sampling for PES/CO with PES ≥ 70% and “Others” bar and circular plots.

In data mining, we found that the dope-dyed garment’s Raman signal was buried under the dye’s fluorescence signal, as shown in Figure 14a, and the piece-dyed garment’s spectrum is in the Figure 14b. The spectrum of the dope-dyed garment exhibits fluorescence signals about five times stronger than those of the piece-dyed garment. To keep the dataset clean and prevent the dope-dyed garment’s spectrum from biasing the training process, we remove them from our dataset.

Data imbalance will highly induce inherent bias while training, and training will tend to the class with a large amount of data. In the textile classification task, having a biased dataset in waste collection is unavoidable. In our collection, two classes are imbalanced: PES/CO with PES ≥ 70% and Others. The small sample of PES/CO with PES ≥ 70% is because of its limited volume in the market [13]. First, we leave the test data out and then perform up-sampling for these two classes, as shown in the histogram in Figure 14c. The test data are used for performance checks in the test stage, while the augmented training set is used for training and validation in the training stage.

After data are preprocessed, the training data are used for training models of machine learning and deep learning. However, with the limited data size, we need to consider the model fitness of our limited training dataset and obtain sound generation while testing in both the under-parameterized machine-learning model and the over-parameterized deep-learning model.

Therefore, we will discuss the training strategy in Section 3.6.1, Section 3.6.2 and Section 3.6.3

### 3.6. Model-Training Strategy

#### 3.6.1. Model Fitness

**Figure 15:**
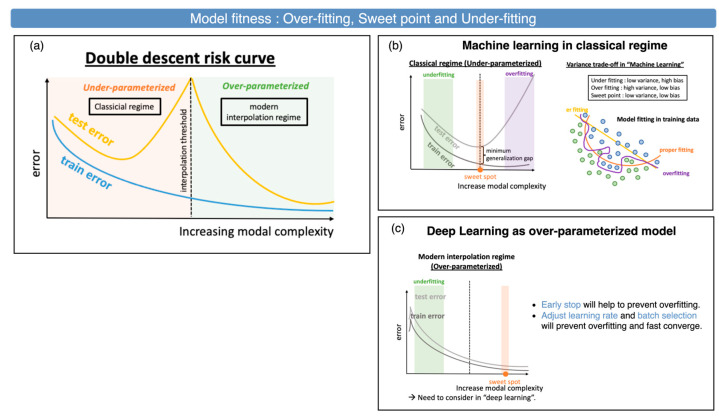
Model fitness for the dataset. (**a**) Double-descent risk curve in the classical ML- and DL-error-curve regime (redrawn based on [47]). (**b**) The classical regime of the ML model’s error curve. (**c**) Over-parameterized DL model’s error curve.

If the model fits the data well, it usually exhibits a lower training error rate in the training process. However, when applied to testing unknown data, there may be a variance in the performance. Hence, a validation set from the training set for the fitted-model check is expected to be split. This is known as the “bias–variance” tradeoff property in machine learning, as shown in Figure 15b. Belkin et al. [47] proposed a double-descent risk curve in 2019, as shown in Figure 15a. It shows evidence that increasing the function class capacity improves the classifier’s performance with no concern for the inductive bias because the function can explicitly or implicitly handle it, as shown in Figure 15c.

#### 3.6.2. Machine-Learning Optimization Strategy

**Figure 16:**
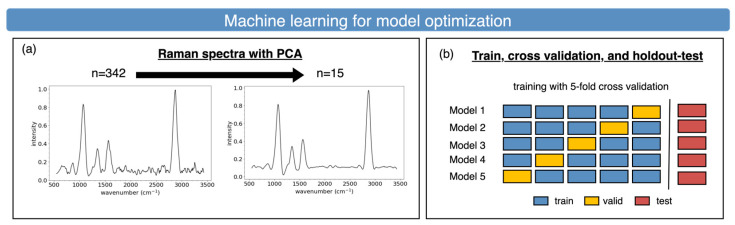

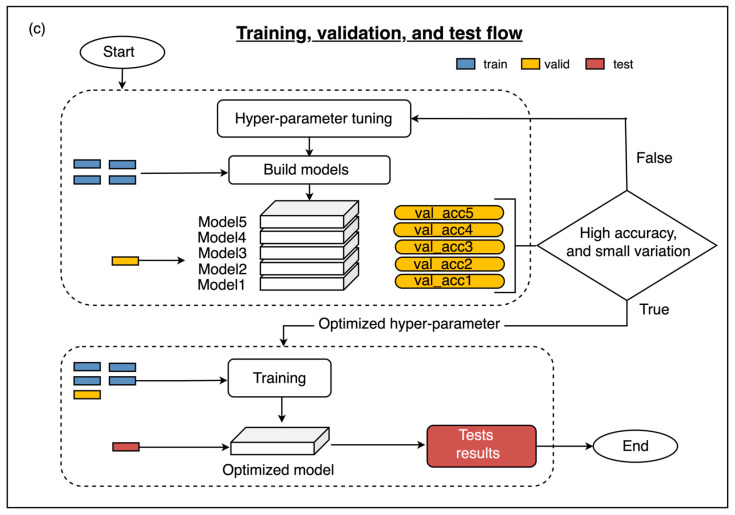
Machine-learning model-training strategies of (**a**) PCA-based dimensionality reduction and (**b**) fivefold cross-validation. (**c**) Training, validation, and test flow.

In machine learning, a bias–variance tradeoff needs to be handled. With the limitation of the training-data size, to fit the under-parameters of the machine-learning model, we reduce the input parameter dimensionality from 342D to 15D with PCA, as shown in Figure 16a. This provides extra benefits for noise reduction and fast inference efficiency. In classical machine learning, cross-validation is commonly conducted to deal with overfitting and underfitting. In our case, we perform fivefold cross-validation for modeling, as shown in Figure 16b, to obtain the “sweet point” (without overfitting and underfitting). The training flow plotted in Figure 16c is used to train a good generalization model by fitting training data to generate the model and test the validation set with a high degree accuracy (in the case of underfitting) and slight variance (in the case of overfitting). This is a recursive process for searching for an optimal under-parameterized machine model that includes KNN, SVM, and RF.

#### 3.6.3. Deep-Learning Optimization Strategy

Recently, deep learning has been proven to be powerful in pattern recognition [48], including image classification [49], recommendation systems [50], and object detection [51] (Zhao Z.Q. et al., 2019). With the double-descent risk curve, the training loss of the over-parameterized deep-learning model will go further after the implementation of a threshold. There are several commonly used strategies for deep-learning model training based on the studies and findings in recent years, not only for 3D image data [42] but also for 1D spectral data [43].

In the deep-learning model optimization, we use the validation set for early stopping [52] once the loss is going to the minimum. The hyperparameter of the learning rate [53] is tuned to make the model converge to the local minimum position. Another hyperparameter, batch size [54], is used to search for the optimal model convergence with an acceptable computational cost.

### 3.7. Model-Training and -Testing Accuracy

#### 3.7.1. Machine-Learning Training and Testing Accuracies

In this section, we compare the performance of three widely used machine learning models—K-Nearest Neighbors (KNN), Support Vector Classifier (SVC), and Random Forest (RF)—based on their training and testing accuracies. Each model was optimized using cross-validation and evaluated on a test set to determine how well it generalizes to unseen data. Below is a detailed evaluation of each model’s performance, including their strengths, weaknesses, and specific challenges encountered during the evaluation process.

- K-Nearest Neighbors (KNNs) [24]

KNN is a well-known machine learning model that classifies an unknown sample based on its nearest neighbors. After optimizing the model using cross-validation, the value of *n* (the number of neighbors) was set to 4. This resulted in a high training accuracy of 97%. However, when evaluated on test data, KNN achieved a testing accuracy of 94%. A notable challenge with KNN is that it tends to misclassify PES/EA textiles as either 100% PES or PES/CO when the polyester content is over 90%, leading to confusion between these categories.

- Support Vector Machine (SVM) [27,28]

The SVM is a machine learning model that works by maximizing the margin between classes using support vectors [55]. We used the radial-basis-function (RBF) kernel with a one-vs-one approach, where each class is compared against others. After tuning the hyperparameters (C = 50,000, and gamma is 0.01) using fivefold cross-validation, we do the model evaluation. The SVM shows strong performance but with some trade-offs in terms of stability and generalization.

- Random Forest (RF) [26]

Random Forest (RF) is a machine learning model that uses multiple decision trees in an ensemble. Each decision trees [25] splits the data based on key features at each node to reduce entropy. To avoid over-fitting, we optimize the *n_estimators* parameter using fivefold cross-validation. The optimized hyperparameters are *max_depth* = 25, and *n_estimators* = 150. Random Forest stands out as the best-performing model, achieving the highest testing accuracy (90.0%) and the lowest variance (0.43%) across 5-fold cross-validation, demonstrating strong generalization and stability.

**Figure 17:**
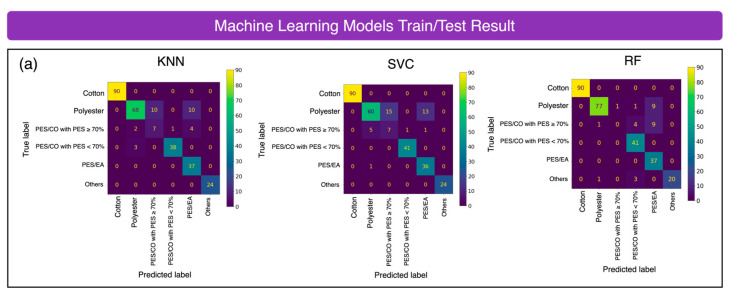

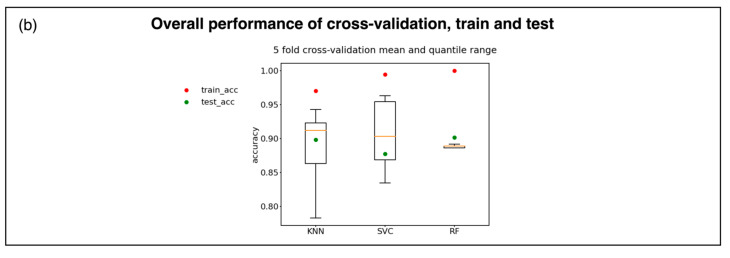
Performance of machine-learning model training and testing. (**a**) The confusion matrix showing the results for three machine learning models: KNN, SVC, and RF. (**b**) The cross-validation box plot, training and testing accuracies of the KNN, SVC, and RF models.

- Machine learning performance comparison:

The confusion matrix in Figure 17a shows how well KNN, RF, and SVC perform in classifying the test data. It highlights the strengths and weaknesses of each model. The box plot in Figure 17b illustrates the variation in accuracy and variance for KNN, RF, and SVC across 5-fold cross-validation, showing how each model performs under different data splits.

SVC achieves the highest validation accuracy (99.0%) but has higher variance (4.9%) as shown in Table 1, suggesting it is less stable across different data splits.

KNN has a high training accuracy (97.0%) but struggles with overfitting, as indicated by its lower testing accuracy (89.5%) and higher variance (5.7%) in Table 1.

Random Forest (RF) delivers the best overall performance with a high testing accuracy (90.0%) and very low variance (0.43%), as shown in Table 1, making it the most consistent and reliable model.

Among these three ML models, Random Forest (RF) stands out as the best model among the three. It balances high accuracy and low variance, making it the most stable and reliable choice for generalization in this task.

#### 3.7.2. Deep-Learning Training and Testing Accuracies

The ANN and CNN models were trained using optimized hyperparameters to efficiently classify Raman spectra, achieving fast convergence without overfitting, as shown in the training/validation curves.

- Artificial Neural Network (ANN)

We used an artificial neural network (ANN) model, as neural networks have been proven to perform well for Raman classification tasks [56]. The architecture of the ANN consists of four fully connected layers, with hidden layers of 128, 64, and 32 neurons. Each hidden layer uses the Mish activation function [57], which introduces nonlinearity to the model. The input spectrum has a dimension of 342, and the output layer has six categories, with a total of 54,438 trainable parameters.

- Learning rate: 0.001;
- Number of learning epochs: 200;
- Batch size: 128;
- Optimizer: Adam [58];
- Convolutional neural network (CNN).

A convolutional neural network (CNN) [32] model was chosen for feature extraction from the Raman spectra. CNNs use a shared kernel that convolves with a sliding window through the data and utilizes backpropagation to optimize the kernels. These learned kernels serve as effective feature extraction tools. CNNs are widely used in image classification, particularly with large datasets, such as CIFAR [59] and ImageNet [60], and have shown strong performance. In our task, we used three layers of 1D CNNs with a kernel size of 5 × 5 and filter numbers of 32, 64, and 128. Each convolution output uses the Swish activation function [61]. The model has a total of 68,198 trainable parameters.

- Learning rate: 0.001;
- Number of learning epochs: 200;
- Batch size: 128;
- Optimizer: Adam [58].

**Figure 18:**
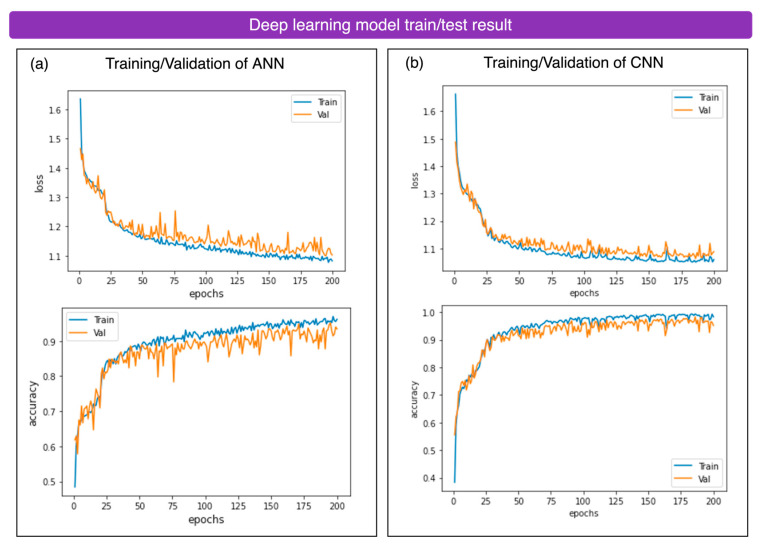
Deep-learning model’s loss and accuracy curves. (**a**) ANN’s training/validation loss and accuracy within 200 epochs. (**b**) CNN’s training/validation loss and accuracy within 200 epochs.

The training and validation curves for both models, shown in Figure 18, demonstrate that both models converge quickly within 200 epochs without overfitting, based on the hyperparameters provided.

**Figure 19:**
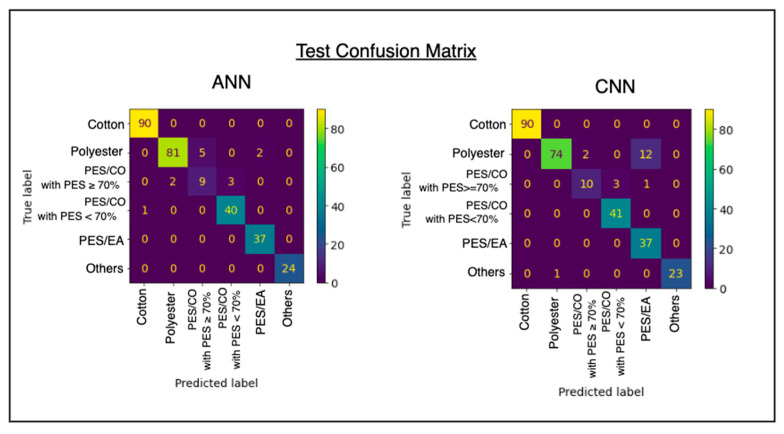
Deep-learning-model confusion matrices of the ANN and CNN with the test dataset.

The confusion matrix in Figure 19 shows that the ANN performs better and has higher accuracy on the test data compared to the CNN model. The CNN model misclassifies some PES/EL as pure polyester, likely because the blended samples contain less than 10% elastane, making them harder for the model to distinguish accurately.

### 3.8. Misclassification Check for the ANN Model

**Figure 20:**
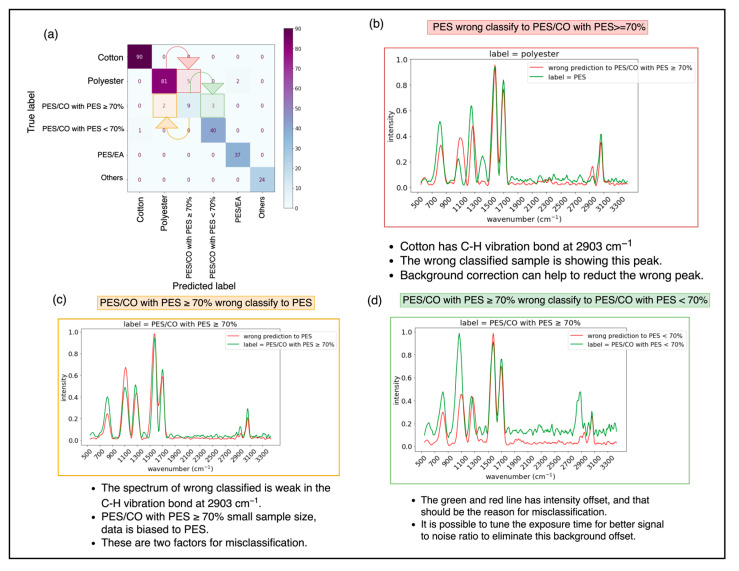
(**a**) Confusion matrix of the ANN model with the test dataset. (**b**) Polyester was wrongly classified as PES/CO with PES ≥ 70%. (**c**) PES/CO with PES ≥ 70% was wrongly classified as Polyester. (**d**) PES/CO with PES ≥ 70% was wrongly classified as PES/CO with PES < 70%.

The confusion matrix for the ANN model, illustrated in Figure 20a, reveals three types of misclassifications:

Five samples were misclassified, where 100% polyester (PES) was incorrectly identified as PES/CO with PES ≥ 70%. This misclassification occurred because of the absence of the C-O bond’s peak at 2903 cm^−1^ in the spectrum, which is characteristic of cotton’s Raman signal (Figure 20b) [62].

Two samples were misclassified from PES/CO with PES ≥ 70% to 100% PES, as shown in Figure 20c. This likely resulted from the limited sample size for PES/CO with PES ≥ 70%, biasing the model toward PES. This issue reflects the scarcity of such textiles in the market.

Similarly, three samples were misclassified from PES/CO with PES ≥ 70% to PES/CO with PES < 70% (Figure 20d). The C-H vibrational bond at 2903 cm^−1^ in spectrum for PES/CO with PES < 70% exhibits a high peak intensity [62], depicted as a green line. The intensity offset between the green and red lines likely caused this misclassification. Improving the signal-to-noise ratio by adjusting the exposure time could help to eliminate this background offset. However, this misclassification is tolerable in the recycling process, as both cases involve chemical recycling and require only minor adjustments to process parameters, ensuring minimal loss in the rPET output ratio [12].

Additionally, two samples of 100% polyethylene (PE) were misclassified as PE/EA. However, advancements in recycling technology, proposed in 2024, allow for effective separation of PE from PE/EA blends, yielding rPET with chemical properties comparable to those of pure polyester. Thus, this misclassification is acceptable within the PE/EA recycling process and maintains reasonable output yield [13].

## 4. Results and Discussion

The accuracies of the three machine-learning (ML) models and two deep-learning (DL) models were evaluated through cross-validation, training, and testing. Among the ML models, The RF exhibited the lowest variance during training. However, all three ML models achieved an approximately 90% testing accuracy. Misclassifications were the most frequent in classes with the lowest data proportions, such as PES/CO with PES ≥ 70% and PES/EA blended textiles. The RF delivered the best performance because of its ensemble nature, which is well suited for classification tasks.

In comparison, The ANN achieved the highest testing accuracy of 96.9%, while the CNN achieved 93.5%, as summarized in Table 2.

The integration of vibrational spectroscopy and AI demonstrates the feasibility of addressing the challenging task of textile material classification, including the classification of blended compositions. The Raman spectra’s high resolution provides detailed vibrational information that AI models can leverage to extract features, map them to the feature domain, and optimize the classification through learned weights.

The results of both classical ML and modern DL models align with the double-descent risk curve phenomenon. In ML models, such as KNN, SVM, and RF, the accuracy exceeds 90%, reflecting a balance between bias and variance. In DL models, such as ANN and CNN, the losses diminish beyond the interpolation threshold, achieving accuracies above 95%. Among the models, the ANN emerged as the most accurate for the proposed Raman-spectroscopy-based Textile Sorter, achieving a 96.9% testing accuracy. The ANN captures global attention through the connections between neurons, whereas the CNN employs local attention via sliding convolutions for feature extraction from Raman spectra.

Table 3 shows the number of correctly classified samples and the classification accuracy by fabric type using the Artificial Neural Network (ANN) model.

The low accuracy for PES/CO with PES ≥ 70% is primarily because of data imbalance in this class. Addressing this limitation through expanded training data collection could enhance the model’s performance. Additionally, PES/CO textiles with PES ≥ 70% are less prevalent in the market, consistent with findings in [13], which reported that Hennes & Mauritz AB (H&M)’s samples included limited PES/CO blends, such as CO/PES = 80/20, 60/40, and 40/60. Despite this, the misclassification is manageable in the recycling process, as both cases involve chemical recycling with minimal adjustments to process parameters, ensuring negligible rPET output loss [12].

Overall, the remaining five fabric classes are classified with a high degree of accuracy, confirming the robustness of the sorting process. This ensures that the subsequent recycling phase achieves a closed-loop flow with high efficiency and purity.

## 5. Conclusions

Currently, without sorting, textile waste faces significant challenges. The majority ends up in landfills or is incinerated, with around 85% of textiles not being meaningfully recycled. Textiles in landfills are non-biodegradable, leaching harmful chemicals into soil and groundwater, and taking up valuable space. Incineration releases toxic pollutants into the air and contributes to climate change, while valuable materials are lost. Both landfill disposal and incineration represent un-recycling processes, as they do not recover or reuse these materials, contributing to environmental harm and resource depletion.

With effective sorting, both closed-loop recycling (fiber to fiber) and open-loop recycling become achievable. Sorting allows for high-quality fiber separation, enabling the recovery and reuse of materials in new textile products (closed loop), or repurposing waste into value-added products, like insulation or construction materials (open loop). This reduces environmental impacts, conserves resources, and supports a more sustainable, circular textile economy.

### 5.1. Raman Spectroscopy with AI for Waste Textile Sorting

We demonstrate a three-fold benefit of integrating AI with Raman spectroscopy to achieve a circular economy:

- High Degree of Accuracy in Fiber Type Classification: Achieving over 95% accuracy in distinguishing between fiber types;
- High Degree of Accuracy in Blend Compositional Analysis: Achieving over 95% accuracy in analyzing blended-fiber compositions;
- High Throughput: Enabling automatic sorting at a speed of one piece per second, replacing manual sorting processes.

Artificial intelligence (AI) implemented through the ANN model in Raman spectroscopy excels in achieving a classification accuracy exceeding 96%. Both machine-learning (ML) and deep-learning (DL) models effectively learn the patterns of textiles’ Raman spectra. Notably, DL models outperformed ML models, with an 8.9% increase in accuracy. Among DL models, the global attention mechanism of the ANN further improved the accuracy by 3.4% compared to that of the local attention mechanism of the CNN. This integrated system enables real-time, high-efficiency, automatic textile sorting, with a Raman signal integration time of one second and a conveyor detection speed of one piece per second.

### 5.2. Data Preprocessing for Enhanced AI Modeling

Data preprocessing is crucial for optimizing Raman spectroscopy’s performance in AI modeling. The key steps include the following:

- Dimensionality Reduction: Enhancing machine-learning models by reducing complexity;
- Balanced Dataset Preparation: Ensuring clean and relatively balanced datasets for effective model training;
- Fluorescence Signal Reduction: Removing dyes’ fluorescence signals to retain only vibrational spectral information essential for AI modeling.

A current limitation of Raman spectroscopy in separating waste textiles is the signal interference caused by dope-dyed fabrics with high concentrations of dark colors [63,64]. However, growing global attention to water pollution caused by dyeing processes [43,65,66] suggests potential solutions. Pretreatment processes for dye removal could mitigate this limitation, facilitating clearer Raman signals for accurate classification.

### 5.3. Achieving Qualitative and Quantitative Sorting with High Degrees of Accuracy and Efficiency

Raman spectroscopy combined with the ANN model enables both qualitative and quantitative sorting with high degrees of accuracy and efficiency. Unlike NIR spectroscopy, which lacks precise quantification, Raman spectroscopy can accurately measure fiber composition and contamination levels. This system supports:

- Closed-Loop Recycling: Sorting purer textiles for recycling into high-quality recycled PES fibers;
- Open-Loop Recycling: Valorizing waste textiles to value-added products, such as wood–plastic composites [14];
- By improving recycling rates and extending textile lifespans, this technology helps to meet the demand for recycled polyester and supports a circular economy.

### 5.4. Future Directions

To further enhance the Raman-spectroscopy-based autoclassification system, future work could explore:

- Transformer Models for Feature Extraction: Implementing transformers [67] in Raman spectroscopy [68] to visualize critical features and understand wavenumber shifts in the spectra of waste textiles;
- Expanding Textiles’ Raman Spectral Datasets: Reducing data imbalance, particularly for textile classes like PES/CO with PES ≥ 70%, through dataset expansion;
- Integrated Background Correction: Utilizing autoencoders, for self-adaptive learning to correct for background interference, seamlessly connected to ANN or CNN networks for one-stage classification.

In conclusion, the integration of Raman spectroscopy with ANNs delivers a powerful solution for waste-textile sorting, combining high degrees of accuracy, efficiency, and adaptability. This technology paves the way for sustainable recycling practices and a robust circular economy for textiles.

## Figures and Tables

**Table 1:** Comparison of KNN, SVC, and RF Models: Training, Validation, and Testing Accuracies.

		KNN	SVC	RF
Five fold cross-validation	Validation accuracy	88.40%	99.00%	100.00%
	Validation STD	5.70%	4.90%	0.43%
Training accuracy		97.00%	90.40%	91.40%
Testing accuracy		89.50%	88.00%	90.00%

**Table 2:** Classification accuracies of three machine-learning models and two deep-learning models.

	KNN	SVC	RF	ANN	CNN
Training accuracy	97.00%	90.40%	91.40%	96.20%	98.00%
Validation accuracy	88.40%	99.00%	100.00%	93.50%	97.00%
Testing accuracy	89.50%	88.00%	90.00%	96.90%	93.50%

**Table 3:** Number of correctly classified samples and classification accuracy by fabric type using ANN model.

Fabric Type	Number of Samples	Number of Samples Correctly Classified	Accuracy of the ANN Model (%)
Cotton (CO)	90	90	100.0%
Polyester (PES)	88	81	92.0%
PES/CO with PES ≥ 70%	14	9	64.3%
PES/CO with PES < 70%	41	40	97.5%
PES/EA	37	37	100.0%
Others	24	24	100.0%

## Data Availability

The data are available upon request.

## Abbreviations

Abbreviation	Definition
3R	reduction, reuse, and recycling
AI	artificial intelligence
ANN	artificial neural network
CE	circular economy
CLR	closed-loop recycling
CNN	convolutional neural network
CO	cotton
DL	deep learning
EA	elastance (spandex)
ESG	environmentally sustainable governance
FTIR	Fourier-transform infrared
IRS	infrared spectroscopy
ITRI	Industrial Technology Research Institute
kNN	k-nearest neighbor
LCA	life cycle assessment
ML	machine learning
NIRS	near-infrared spectroscopy
OLR	open-loop recycling
PC	principal component
PCA	principal component analysis
PES	polyester
PES/CO	polyester and cotton blend
PES/EA	polyester and elastance blend
PA	polyamide
RF	random forest
rPET	recycled polyethylene terephthalate
S-G filter.	Savitzky–Golay filter
SVM	support vector machine
